# Genealogical relationship inference to identify areas of intensive poaching of the Orange-fronted Parakeet (*Eupsittula canicularis*)

**DOI:** 10.1186/s40850-021-00080-y

**Published:** 2021-05-10

**Authors:** Gabriela Padilla-Jacobo, Tiberio C. Monterrubio-Rico, Horacio Cano-Camacho, María Guadalupe Zavala-Páramo

**Affiliations:** 1 Centro Multidisciplinario de Estudios en Biotecnología, FMVZ, Universidad Michoacana de San Nicolás de Hidalgo, Km. 9.5 Carretera Morelia-Zinapecuaro, Posta Veterinaria, Morelia, Michoacán Mexico; 2 Laboratorio de Ecología de Vertebrados Terrestres Prioritarios, Facultad de Biología, Universidad Michoacana de San Nicolás de Hidalgo, Edificio R, CU, Morelia, Michoacán Mexico

**Keywords:** Illegal trade, Cytochrome b, Genetic diversity, Population expansion, Phylogeography

## Abstract

**Background:**

The Orange-fronted Parakeet (*Eupsittula canicularis*) is the Mexican psittacine that is most captured for the illegal pet trade. However, as for most wildlife exploited by illegal trade, the genetic diversity that is extracted from species and areas of intensive poaching is unknown. In this study, we analyzed the genetic diversity of 80 *E. canicularis* parakeets confiscated from the illegal trade and estimated the level of extraction of genetic diversity by poaching using the mitochondrial DNA sequences of cytochrome b (Cytb). In addition, we analyzed the genealogical and haplotypic relationships of the poached parakeets and sampled wild populations in Mexico, as a strategy for identifying the places of origin of poached parakeets.

**Results:**

Poached parakeets showed high haplotype diversity (Hd = 0.842) and low nucleotide diversity (Pi = 0.00182). Among 22 haplotypes identified, 18 were found exclusively in 37 individuals, while four were detected in the remaining 43 individuals and shared with the wild populations. A rarefaction and extrapolation curve revealed that 240 poached individuals can include up to 47 haplotypes and suggested that the actual haplotype richness of poached parakeets is higher than our analyses indicate. The geographic locations of the four haplotypes shared between poached and wild parakeets ranged from Michoacan to Sinaloa, Mexico. However, the rare haplotypes detected in poached parakeets were derived from a recent genetic expansion of the species that has occurred between the northwest of Michoacan and the coastal region of Colima, Jalisco and southern Nayarit, Mexico.

**Conclusions:**

Poached parakeets showed high genetic diversity, suggesting high extraction of the genetic pool of the species in central Mexico. Rarefaction and extrapolation analyses suggest that the actual haplotype richness in poached parakeets is higher than reflected by our analyses. The poached parakeets belong mainly to a very diverse genetic group of the species, and their most likely origin is between northern Michoacan and southern Nayarit, Mexico. We found no evidence that poachers included individuals from Central American international trafficking with individuals from Mexico in the sample.

**Supplementary Information:**

The online version contains supplementary material available at 10.1186/s40850-021-00080-y.

## Background

Illegal exploitation of natural resources is the third most profitable criminal business in the world [1]. Within wildlife crime, illegal logging, illegal fishing, and illegal wildlife trade account for between $72.5 and $216.4 US billion annually [1]. Poaching and illegal trade involve a multitude of floral and faunal species, a variety of markets and a complex network of operators [1, 2]. This activity causes direct damage to biodiversity and can lead to the extinction of populations of many species, among other serious implications. International organizations such as the Convention on International Trade in Endangered Species of Wild Fauna and Flora (CITES), United Nations Office on Drugs and Crime (UNODC), International Union for Conservation of Nature (IUCN), and World Wildlife Fund (WWF), among others, have worked to discourage these practices. Despite these efforts being reinforced by the wildlife protection laws of various countries, the practices persist.

Globally, the order Psittaciformes dominates the pet bird trade; its market demand has caused all species of the order to be listed in the CITES protected species appendices (except four common species) [3, 4]. The illegal trade in parrots is of great concern in Latin America. From 2007 to 2014, New World parrots of the *Eupsittula* and *Aratinga* genera remained in second place in parrot trafficking detected through international customs [3]. Brazil, Mexico, and Peru have reported parrot seizures in their countries [3]. However, due to the complex network of operators, the origin and final market of these birds remains unknown.

In the case of psittacines in Mexico, illegal trade is one of the main factors causing the decline of parakeet populations [5–8]. In this country, the illegal parrot trade supplies national and international markets, where they are sold as pets [5, 7]. According to estimates of the number of individuals captured and mortality rate, between 50,050 and 60,445 individuals die each year from poaching [7]. After confiscation, specimens are confined under quarantine in state zoos or in Wildlife Research and Conservation Centers (CIVS, Spanish acronym). The primary purpose of these centers is to rehabilitate and release confiscated parrots; however, the birds generally remain in zoos indefinitely because their place of origin is unknown. Although some routes are known to be used by poachers to transport parakeets [7], determining their origin is particularly difficult because parakeets from different regions are confined together, so the origin and routes are uncertain. In addition, poachers capture chicks from the nests in the wild before they can fly [3, 7]. Therefore, these individuals have not reached sexual maturity, have no offspring, and so have not contributed to the gene pools of their populations before being captured; in other words, populations have been unable to recruit breeders, reducing their ability to support themselves, while reducing their genetic diversity. Most of the time, the genetic diversity of the confiscated parrots and the impact of their poaching on the gene pool of the species is unknown.

**Fig. 1:**
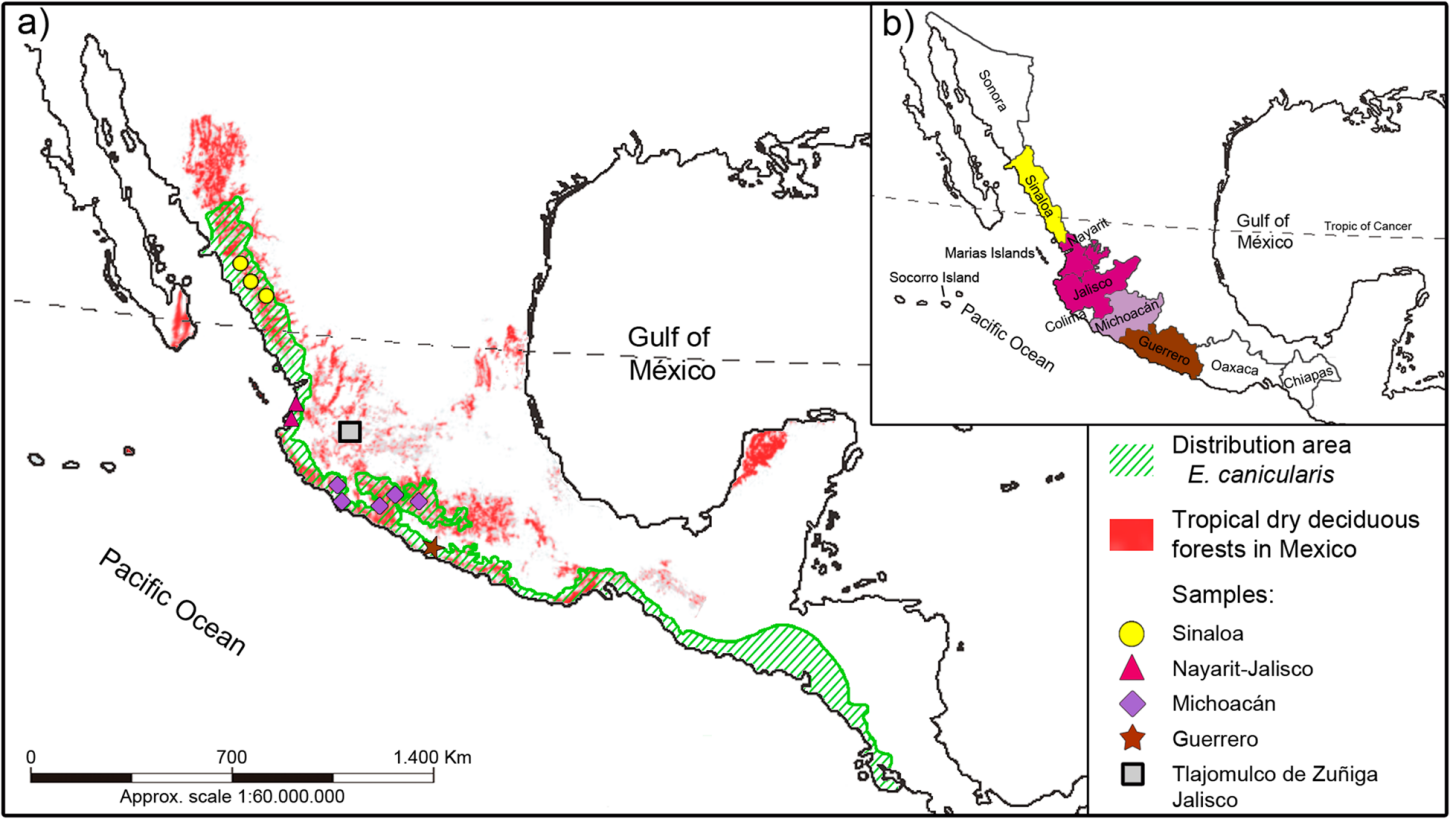
Geographic distribution and sampling regions of *E. canicularis*. **a** The red shaded area is the tropical dry deciduous forest in Mexico one of the main habitats of the species. The striped green area represents the potential distribution of the species according to Monterrubio-Rico et al. [14] and Collar et al. [16]. The box indicates the location where the parakeets were confiscated (Tlajomulco de Zuñiga, Jalisco, Mexico). **b** Map of the political divisions of Mexico showing the states on the Pacific slope with the collection regions for wild populations of *E. canicularis.* The map was constructed using the potential distribution data of the species [14, 16] and free public consultation vegetation layers available at https://www.inegi.org.mx/app/mapa/espacioydatos/?ly=1456.1450.1451.1452.1453.1454.1455.1457.1458.1459.1460.1461.1462.1507, with the Adobe Photoshop CS5.1 software

The Orange-fronted Parakeet (*E. canicularis*) inhabits the Pacific slope from northern Mexico to northern Costa Rica [9–11] (Fig. 1). The habitat of the species includes moist and subhumid deciduous forests, tropical dry deciduous forests, riparian forests, and agricultural areas [10–13]. Based on fieldwork and ecological niche models, the actual range with suitable ecological conditions is estimated to cover 247,312 km^2^ along the Pacific coast of Mexico, with a low percentage of distribution inside protected areas (1.6%) [14]. Unfortunately, the decline in populations of highly demanded psittacine species also occurs within conserved forest fragments. In areas where the intensity of poaching is high, extirpations of local populations occur, further reducing the recruitment potential of regional populations, as evidenced for *Amazona oratrix* and *Ara militaris* in the central Pacific slope [7, 15].

*E. canicularis* is affected by the highest degree of extraction for the illegal parrot trade in Mexico, due to its wide distribution and the low height of the termitaria where parakeets nest [5, 7]. The Official Mexican legislation NOM-059 [17] lists *E. canicularis* in the special protection category, which includes species or populations that could be threatened by factors that negatively affect their viability and, therefore, it is necessary to promote their recovery and conservation. One factor that affects the viability of the species is poaching. Between 2017 and 2019, the Mexican authorities confiscated 2012 *E. canicularis* parakeets from illegal trade [18]. The extent to which poaching affects population trends and viability is unknown, as the actual population size of the species in Mexico is difficult to determine, because population abundance data are not available for each region [9]. Studies from 2005 to 2008 in the central Pacific slope revealed that the abundance of *E. canicularis* varies by forest type, the proportion of primary habitat in the landscape, and the historical intensity of poaching. On the Jalisco coast, *E. canicularis* was the most abundant psittacine species, represented by 76% (430 out of 657) of the individuals recorded among four species in a point count balanced design study. The apparent density estimates derived from this design corresponded to 86 parakeets/km^2^ [19]. In three coastal Michoacan landscapes with intense poaching pressure, the relative abundance of this species was only 31% (466 out of 1480 individuals) in a balanced study design with six survey areas covering 1.5 km^2^ with an average of 6.4 parakeet/km^2^ [20].

Recently, genetic diversity, intraspecific divergence, and demographic history of *E. canicularis* were evaluated through molecular analysis of biological samples from individuals from wild populations distributed on the Pacific slope of Mexico from Sinaloa to Guerrero [21]. A high genetic diversity and three genetic groups with overlapping geographic distributions on the north coast of Michoacan were identified based on Cytb sequence analysis of 53 georeferenced individuals. In addition, a population expansion of the species was determined to have occurred during the Upper Pleistocene, through the analysis of the 53 parakeets from wild populations and 80 confiscated parakeets from illegal trade. In particular, it was found that the wild populations distributed in the north exhibited lower diversity than the populations distributed towards central Mexico [21]. Therefore, poaching may be more detrimental to northern populations with lower diversity than to populations with higher diversity in central Mexico.

Therefore, the geographic contraction of populations and their numerical declines can lead to genetic diversity loss, inbreeding, reduced reproductive fitness (inbreeding depression) and reduced ability to evolve in response to environmental change [22]. Under the recruitment limitations imposed on the local population by the illegal trafficking of parakeet chicks, the occurrence of such a process depends on the level of genetic diversity of the species, the size of the population and the proportions of generations of chicks removed by poaching. Identifying core areas from which a high number of chicks were trafficked is essential, as it provides the basis for conservation actions such as poaching control and population reintroductions of confiscated parakeets, as well as wildlife sanctuary designation.

Forensic science provides a range of tools to wildlife law enforcement officers worldwide [23–25]. Uncertainty regarding crimes committed in the illegal wildlife trade can be resolved using techniques based on DNA polymorphism. Some of the issues that can be solved through forensic genetics are species identification, its geographical origin, individual identification, sexing and paternity [23–25]. In wildlife forensic science, investigation of each crime victim species is necessary to validate their routine use in forensic application and admissibility as evidence [25]. In the illegal wildlife trade, a large number of species lack genetic data to help resolve uncertainties that arise when these crimes are committed. Currently, in Mexico, wildlife forensic science research applied to psittacines is practically nonexistent.

In the present study, we performed the first analysis of the genetic diversity of 80 poached *E. canicularis* parakeets and estimated the level of extraction of genetic diversity by poaching through rarefaction and extrapolation curves using Cytb sequences. In addition, we identified the likely places of origin of poached parakeets through the detection of haplotypes shared between individuals from wild populations and poached birds, the analysis of haplotype relationships and genealogical analysis.

## Results

### Genetic diversity

The sequence matrix analyzed to examine the genetic diversity of poached parakeets included 80 sequences and 931 characters. The data revealed 22 haplotypes with 904 invariant sites and 27 polymorphic sites, of which 15 were singleton variable sites and 12 were parsimony informative sites (Additional file 1). The mutations included 23 transitions and four transversions; indels were not observed. A high haplotype diversity (Hd = 0.842 +/− 0.033) and low nucleotide diversity (Pi = 0.00182 +/− 0.00119) were detected (Table 1). The composition of bases was adenine (A) 26.83%, thymine (T) 25.12%, cytosine (C) 35.03%, and guanine (G) 13.02%.

**Table 1:** Genetic diversity indices in Cytb gene from *E. canicularis* samples

Group	Samples (n)	Alignment	H	Hd (SD)	Pi (SD)	S
Poaching	80	931	22	0.842 (+/− 0.033)	0.00182 (+/−0.00119)	27
Wild population	53	931	18	0.879 (+/−0.026)	0.00222 (+/−0.00140)	25
Total	133	931	36	0.878 (+/−0.022)	0.00206 (+/−0.00131)	44

*n* number of individuals, *H* number of haplotypes, *Hd* haplotype diversity, *SD* Standard deviation, *Pi* Nucleotide diversity, *S* Polymorphic sites

The analysis of 53 sequences and 931 characters from the wild populations showed 18 haplotypes with 906 invariant sites and 25 polymorphic sites, of which 16 were singleton variable sites and nine were parsimony informative sites (Additional file 1). The mutations included 24 transitions and one transversion; indels were not observed. A high haplotype diversity (Hd = 0.879 +/− 0.026) and low nucleotide diversity (Pi = 0.00222 +/− 0.00140) were detected (Table 1). The composition of bases was A = 26.82%, T = 25.10%, C = 35.05%, and G = 13.03%.

When the sequences from wild populations and those of the poached parakeets were analyzed together, the resulting data revealed 36 haplotypes with 887 invariant sites and 44 polymorphic sites, of which 25 were singleton variable sites and 19 were parsimony informative sites (Table 1). The mutations included 39 transitions and five transversions; indels were not observed. A high haplotype diversity (Hd = 0.878 +/− 0.022) and low nucleotide diversity (Pi = 0.00206 +/− 0.00131) were detected (Table 1). The composition of the bases was A = 26.83%, T = 25.11%, C = 35.04%, and G = 13.02%.

**Fig. 2:**
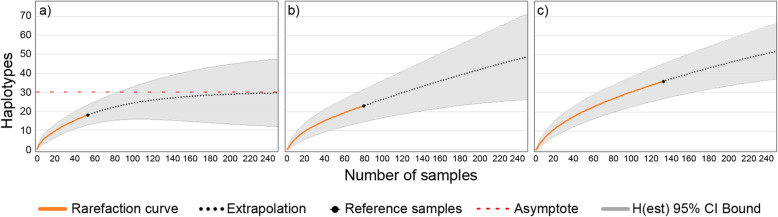
Rarefaction and extrapolation curves based on Cytb data showing the expected numbers of haplotypes in three groups of *E. canicularis* samples*.* **a** Estimation of the number of haplotypes in wild parakeets. **b** Estimation of the number of haplotypes in poached parakeets. **c** Estimation of the number of haplotypes in wild + poached parakeets. H (est): Estimated number of haplotypes

Because it is not possible to collect and analyze biological samples from all individuals in the illegal trade to estimate the level of extraction of genetic diversity from the natural population of *E. canicularis* by poaching, we applied a methodology frequently used in ecological analysis. Rarefaction and extrapolation curves estimated with the 22 haplotypes of the 80 poached parakeets showed the mean of the expected richness values, and a slight but constant growth proportional to the number of samples (individuals) that did not reach the asymptote within 240 samples (Fig. 2b). The curves show that there would be approximately 36 haplotypes (95% confidence interval CI = 21.76–50.29) in a group of 160 individuals, a number of haplotypes similar to that detected for the total number of poached and wild parakeets analyzed (36 haplotypes in 133 individuals), and 47 haplotypes (95% CI = 25.95–68.64) for a group of 240 individuals (Fig. 2b). When extrapolation was performed to 320 individuals, the CI range was broadened and became less reliable (mean = 57. 95%, CI = 27.34–86.76, not shown in graph). Furthermore, the rarefaction and extrapolation curves estimated with the 36 haplotypes of the 133 individuals (wild and poached) revealed a trend similar to that observed for haplotype simulations of poached parakeets. In this graph, the curves show that there would be approximately 40 haplotypes (95% CI = 29.85–50.34) in a group of 160 individuals and 50.52 haplotypes (95% CI = 36.48–64.55) for a group of 240 individuals (Fig. 2c). However, the estimates made with the 18 haplotypes of the 53 wild parakeets did not reach an asymptote with the number of samples analyzed (Fig. 2a); in the extrapolation, the asymptote was reached within approximately 304 individuals (not shown in graph), with an estimate of the number of haplotypes equal to 29 (95% CI = 10.98–49.02) (Fig. 2a).

### Haplotype relationships

**Fig. 3:**
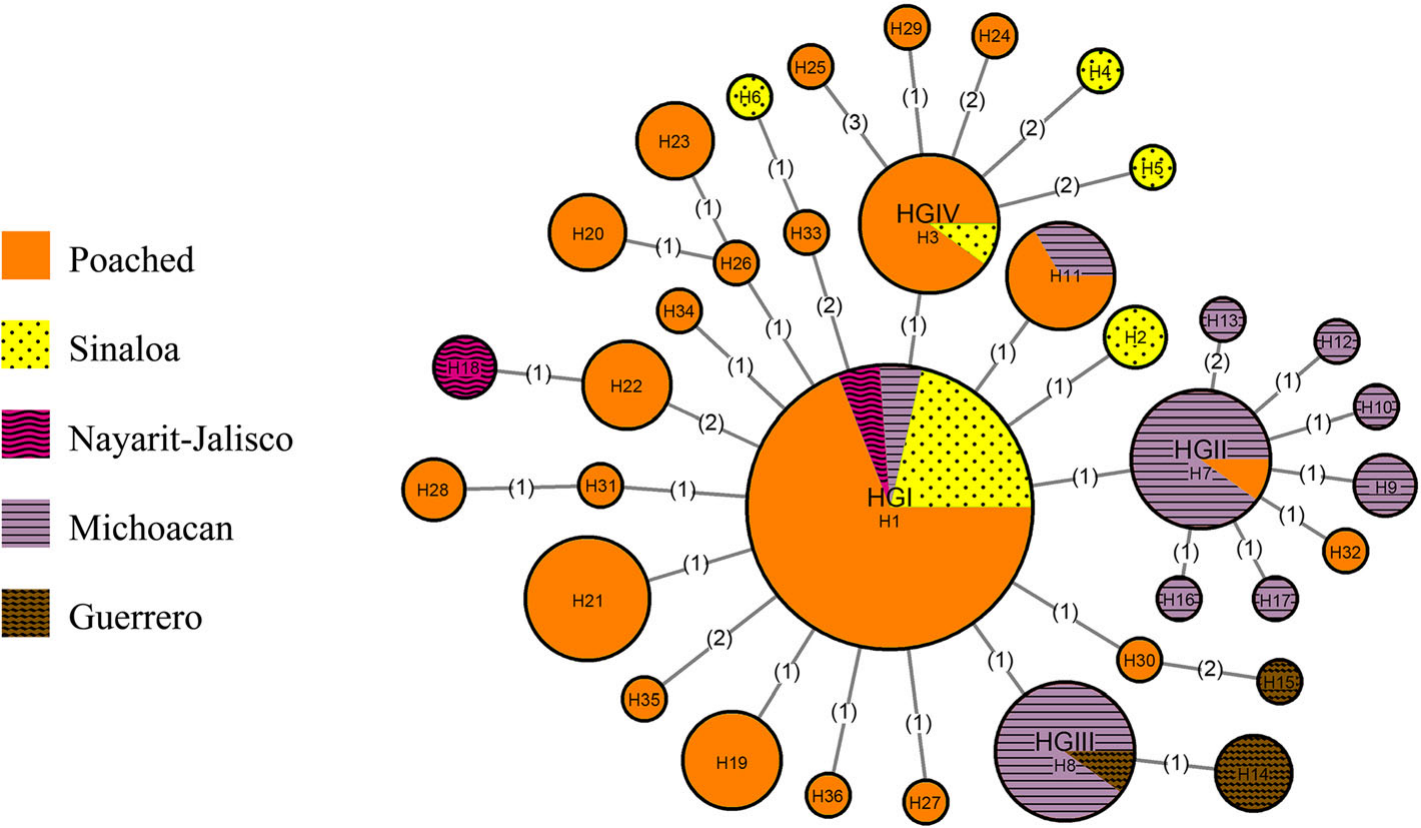
Median-joining haplotype network illustrating the relationships among 36 haplotypes from 133 individuals of *E. canicularis* based on Cytb. Circle size is proportional to haplotype frequency; branch length is not proportional to the number of mutations. Numbers on the branches represent mutations between haplotypes. HGI, HGII, HGIII and HGIV correspond to haplogroups I, II, III, and IV, respectively

In the haplotype network constructed with 36 haplotypes and 133 individuals, a maximum of three mutational steps was observed among the haplotypes. The network showed the typical star shape of expanding populations, consistent with Tajima’s D value (− 2.326, *p* < 0.001) and Fu’s F value (− 27.536, *p* < 0.0001) [21, 26–28], with four haplogroups distinguished (Fig. 3). In haplogroup I (HGI), a central haplotype (H1) was observed with 16 directly connected peripherals, of which 11 haplotypes were exclusively of poached parakeets. In haplogroup II (HGII) the central haplotype H7 was shared by poached parakeets and those from the wild population near the Michoacan coast (Figs. 1 and 3). Haplogroup III (HGIII) with the central haplotype H8 included parakeets from wild populations of Michoacan and Guerrero (Figs. 1 and 3) but not from poaching. Finally, in haplogroup IV (HGIV) the central haplotype H3 was found mainly in poached parakeets with some instances from the wild population of Sinaloa, and their peripheral haplotypes were from poached parakeets and the wild population of Sinaloa (Figs. 1 and 3).

The haplotypes H1 (*n* = 29), H3 (*n* = 9), H11 (*n* = 4), and H7 (*n* = 1) were shared by 43 poached parakeets (54%) and those from wild populations. In addition, 18 haplotypes not previously found in wild populations were detected in 37 poached parakeets (Fig. 3, Additional file 1).

### Genealogical analysis

**Fig. 4:**
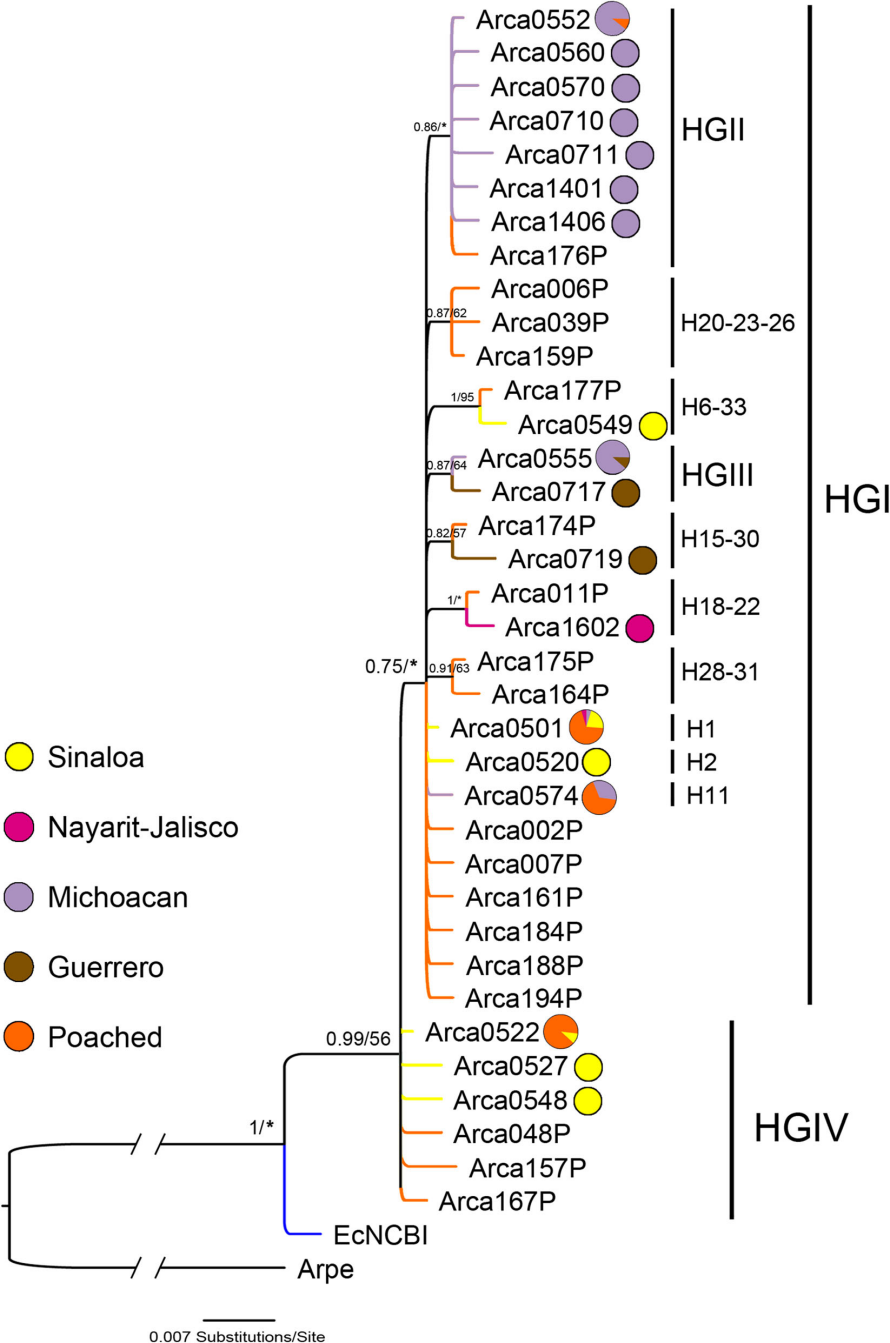
Consensus tree showing the genealogic relationships among haplotypes of *E. canicularis,* obtained using Bayesian inference (BI) and maximum likelihood (ML) analyses. Estimates were made with 931 characters from 37 haplotypes of Cytb. The out group is the sister species *Eupsittula pertinax*. Values on the branches represent posterior probabilities and bootstrap values (PP/BP). (*) Value inferior at PP = 0.5 or BP = 50. The branches-support values were estimated using a bootstrap analysis (BP) of 500 replicates and posterior probabilities (PP) for 10 million generations, sampling one tree every 1000 generations

The matrix for the genealogical analysis included the 36 haplotypes with 931 characters. There where 44 polymorphic sites detected, of which 33 were singleton variable sites and 11 were parsimony informative sites. We included a Cytb sequence reported for a specimen identified as *E. canicularis* (EcNCBI hereinafter) (GenBank Access: KJ142251.1). In addition, a sequence of *Eupsittula pertinax* was included as an outgroup (GenBank Access: HM640208.1). The consensus tree built via ML and BI is illustrated in Fig. 4. In both analyses the same topology was observed. Although some nodes do not have high PP or BP values, the presented topology allows us to review the genealogic relationships of the detected haplogroups. In particular, the formation of clades allowed the identification of haplotypes not shared between parakeets from wild populations and poached parakeets. The EcNCBI haplotype revealed early divergence from the rest of the analyzed haplotypes.

In the consensus tree, although a smooth polytomy is observed, it is possible to detect that a group of haplotypes from the wild population of Sinaloa (Fig. 1) and from the poached parakeets belonging to the HGIV haplogroup are ancestors of the rest (Figs. 3 and 4). The HGI clade contains haplotypes from wild populations of Sinaloa, Nayarit, Jalisco, Michoacan, and Guerrero (Figs. 1 and 4). This clade presents a polytomy that includes the H1 haplotype and its derivatives belonging to the HGI haplogroup, as well as some particularly interesting subclades. The HGII subclade corresponds to the haplogroup present in the wild population from the Michoacan coast (Figs. 1, 3 and 4). In this subclade, the haplotypes of the poached parakeets Arca0552/Arca026P (shared) and Arca176P are closely related to seven haplotypes from the wild population. The HGIII subclade corresponds to the haplogroup of the wild populations of Michoacan and Guerrero without poached parakeets (Figs. 1, 3 and 4). The H20–23-26, and H28–31 subclades only have haplotypes of poached parakeets, derived from the dominant H1 haplotype of the HGI haplogroup (Figs. 3 and 4). Two individuals from the wild population of Michoacan and four poached parakeets shared the H11 haplotype (Figs. 1, 3 and 4). The H18–22 subclade has a haplotype shared by two individuals from the wild population of Jalisco, derived from another haplotype shared by four poached parakeets, derived in turn from the H1 haplotype (Figs. 1, 3 and 4). Two individuals from the wild population of Sinaloa shared the H2 haplotype (Arca0520). The H6–33 subclade has haplotypes derived from the H1 haplotype, one from the wild population of Sinaloa and one from a poached individual (Figs. 1, 3 and 4). The H15–30 subclade includes a haplotype from the wild population of Guerrero and one poached parakeet, both derived from the H1 haplotype (Figs. 1, 3 and 4).

## Discussion

Knowledge of the geographic origin of individuals can be used to distinguish legal and illegal products, assist in the repatriation of poached parakeets and identify which parakeets are most heavily exploited for trade [25]. The geographic origin of the individuals can be identified as long as there is a known genetic structure within the region of interest [25]. In the present case, due to previous work on the phylogeography of *E. canicularis*, we were able to identify the most likely area of poaching.

According to Grant and Bowen [28], the Hd values above 0.5 are considered high, and those below 0.5 are considered low. In birds, low levels of genetic diversity have been reported in species that have gone through bottlenecks (*Gymnogyps californianus*, Hd = 0.543 (*n* = 207) [29]; *Calidris canutus*, Hd = 0.69 (*n* = 25) [30]; *Pseudonestor xanthophrys*, Hd = 0.382 (*n* = 85) [31]). On the other hand, high levels of Hd have been associated with recent population expansion (*Lepidocolaptes affinis*, Hd from 0.00 (*n* = 3) to 0.95 (*n* = 15) [32], *Ergaticus ruber*, Hd above of 0.943 (*n* = 58) [33]; *Lampornis amethystinus* Hd = 0.91 (*n* = 69) [34]). In psittacines, there are some varied examples; some populations of *Amazona leucocephala* have Hd values ranging from 0.46 (*n* = 8) to 0.69 (*n* = 25) [35]), in *Amazona aestiva* shows a range of 0.0 (*n* = 3) to 0.78 (*n* = 13) [36]), and for *Anodorhynchus hyacinthinus* the values range from 0.182 (*n* = 24) to 0.831 (*n* = 19) [37]). Genetic diversity is known that to be necessary for populations to adapt to environmental changes, and the loss of genetic diversity reduces evolutionary potential and is also associated with reduced reproductive fitness [22]. The results of genetic diversity found in our study indicate a high haplotypic diversity; in wild parakeet populations (Hd = 0.879, *n* = 53), this may indicate that the metapopulation still has retains the ability to recover from the impact of past poaching. For poached parakeets (Hd = 0.842, *n* = 80), this information can be used as reference for the design of conservation strategies; for example, to avoid the negative consequences of population inbreeding in protected areas, or to avoid bottleneck or founder effects in released and/or recipient populations.

Of 36 haplotypes detected in the 133 individual wild and poached parakeets, 22 haplotypes were founded exclusively in poached parakeets. Poaching captured mainly individuals of the H1 haplotype (*n* = 29), followed by the H3 (*n* = 9), H11 (*n* = 4), and H7 (*n* = 1) haplotypes (Figs. 3 and 4), providing further information on the frequencies of these haplotypes and revealing the likely originating geographical distribution of these poached parakeets from the wild populations of the species. On the other hand, some poached parakeets revealed 18 exclusive haplotypes, of which 12 were unique in 12 individuals. We believe that the species may lose genetic diversity if individuals with rare and low-frequency haplotypes are eliminated in the short to medium term, as a consequence of the poaching of thousands of individuals each year.

The results of the rarefaction and extrapolation curves using the data set of poached parakeets showed an estimate of the richness of haplotypes in the population of the species, close to that detected in the total number of poached and wild parakeets analyzed (*n* = 133) and suggesting that poaching of 240 individuals can extract up to 47 haplotypes (Fig. 2). In other words, because the analyses with 80 (poached) and 133 individuals did not reach the asymptote, it is suggested that the actual haplotype richness in poached parakeets is higher than reflected in our analyses.

Our results suggest that poached parakeets originate from the wild population of central Pacific slope of Mexico. The wild population of *E. canicularis* distributed from Guerrero to Sinaloa (Fig. 1) has a high genetic diversity and three genetic groups with overlapping geographic distribution on the north coast of Michoacan [21]. However, wild individuals belonging to two genetic lineages in central Pacific slope of Mexico previously showed higher genetic diversity than the individuals in the genetic lineage of the north. Thus, it was established that the H1, H3, H7, and H11 haplotypes detected in 43 poached parakeets belong to a genetic group distributed from Michoacan to Sinaloa (Fig. 1) [21].

The genetic groups of *E. canicularis* in central Pacific slope of Mexico include private haplotypes due to in situ diversification during the recent expansion of the species in the Upper Pleistocene [21]. In the topology of haplotype networks, recent expansion processes from a single geographic source (i.e., diversification in situ) are observed as starburst patterns (Fig. 3) where the common and widespread haplotype is probably the ancestral condition from which rare (private) haplotypes have recently been derived [27, 38, 39]. In particular, the HGII haplogroup is distributed only in its area of origin (Michoacan), while the HGI haplogroup expanded in range from central to northern Mexico (Figs. 3 and 4). Similar processes have been identified in *Meleagris gallopavo*, where certain haplotypes of a genetic lineage from approximately 1.62 million years ago (Mya) settled in their place of origin in the northwest of what is currently the USA, founding a population that does not undergo range expansion, while other haplotypes of the same genetic lineage continued to diversify and expand their range until reaching central Mexico [40].

The H7 and H11 haplotypes have been found in the wild population of Michoacan in particular (in the coastal region and Balsas basin, respectively) (Figs. 1 and 3) [21], suggesting that poached parakeets with these haplotypes belong to this region. In addition, since H22, H30, H32, and H33 haplotypes detected in seven poached individuals were peripheral to the H1 and H7 haplotypes (corresponding to HGI and HGII haplogroups, respectively) (Figs. 3 and 4) and were included in subclades with haplotypes from the wild populations of the Michoacan coast (HGII), Guerrero coast (H15–30), Nayarit-Jalisco (H18–22), and Sinaloa (H6–33) (Figs. 1, 3 and 4), we propose that poached parakeets with these haplotypes have their southernmost area of origin in northwestern Michoacan (Fig. 1).

On the other hand, the H1 haplotype was found mainly distributed in wild populations of Sinaloa, with lower proportions in Nayarit, Jalisco, and Michoacan and was not detected in the localities of Guerrero [21]. However, the H1 haplotype is the ancestor of a large number of peripheral haplotypes detected in poached parakeets and wild population, has the highest frequency, and is distributed from central to northern Mexico (Figs. 3 and 4), suggesting that it coexists with its derived haplotypes in Michoacan, Colima, Jalisco, Nayarit, and Sinaloa (Fig. 1).

The H3 haplotype was detected in nine poached parakeets, previously showing a distribution only in the wild population of Sinaloa [21]. However, in the tree topology and the haplotype network, this haplotype is the ancestor of the H1 haplotype by a mutation (Figs. 3 and 4), suggesting that the H3 haplotype and its derived haplotypes, including the H25, H29, and H24 haplotypes detected in poached parakeets, are distributed from central to northern Mexico (Fig. 1). Thus, considering that most of the haplotypes of poached parakeets were found in central Mexico, we propose that parakeets with the H25, H29, and H24 haplotypes were also captured in the same region.

In addition, no shared haplotypes were detected between poached parakeets and wild population located in Guerrero, where a second genetic group of the species was detected [21]. Since poached parakeets belong mainly to one of the three genetic groups previously identified in the species, they thus originated in the region between northern Michoacan and southern Nayarit (Fig. 1). Therefore, our results refute the statement of the vendor that all parakeets were captured in Sinaloa, Mexico.

On the other hand, genealogical analysis showed that the EcNCBI haplotype is the ancestor of the rest of the analyzed haplotypes (Fig. 4), corroborating a similar relationships for which a separation by 10 mutational steps was detected [41]. However, it should be noted that the EcNCBI haplotype shows a close relationship with the HGIV haplogroup (Figs. 3 and 4). The genetic distance of the EcNCBI haplotype demonstrates a certain degree of isolation, which could be the result of geographical distance. If this is the case, we assume that it originated in locations in the south beyond our sampling, including Mexico or Central America. Under this assumption we suggest that the poached individuals do not come from populations in southern Mexico or Central America. Ancestral haplotypes are in Central America and the most recent haplotypes are in Mexico. This implies that in the topology of genealogical trees the ancestral haplotypes, such as the EcNCBI haplotype, are basal (Fig. 4). Therefore, its geographical distribution may be at or near the place of origin of the species, because it settled in the area without range expansion. Furthermore, even if ancestral haplotypes were distributed from the south to the north in Mexico due to a range expansion process, they would be basal in genealogical trees. Thus, haplotypes with southern distributions must be basal in genealogic trees, and in this case must be closely related to the EcNCBI haplotype by a few mutations. Therefore, we assume that the EcNCBI haplotype may correspond to an individual from a population distributed to the south in Mexico or Central America. In addition, since this haplotype or one closely related to it were not found in poached parakeets, this suggests that they do not come from populations in southern Mexico or Central America.

Cantú-Guzmán et al. [7] described how local poachers in Nayarit state sell the captured parakeets to traders in Guadalajara. The results of this study corroborate that poaching and sales in the domestic market include parakeets from different locations in different states and not from one locality. Our data support the conclusion that poached parakeets from different locations in a region are held together for subsequent sale, a previously reported practice carried out by captors and merchants [7]. The poaching range established in the sample examined extends from Nayarit to the north of Michoacan, where the number of poachers and operators involved is unknown. We found no evidence that poachers mixed individuals from international trafficking with the individuals analyzed in this study; in this group, only closely related haplotypes were detected. The evidence provided by our analysis will allow continuing with investigations to clarify crime routes and operators. Additionally, since parrots dominate the pet bird trade, our data can be helpful in identifying whether parakeets seized in international customs originate from central and northern coast of Mexico.

Databases provided by molecular analysis after sampling constitute one of the most valuable sets of information for conservation purposes. Estimates of genetic diversity could be applied for genetic restoration and the establishment of the captive-bred colonies. For example, if the goal is to release individuals to strengthen wild populations, the organisms evaluated are suitable if they show high genetic diversity. On the other hand, when designing a captive breeding program, the genotypes of individuals should be considered with the aim of increasing genetic diversity [42–46].

## Conclusions

Poached *E. canicularis* parakeets showed high genetic diversity, suggesting high extraction from the genetic pool of the species in central Mexico. Rarefaction and extrapolation analyses suggest that the actual haplotype richness in poached parakeets is higher than our analyzes indicate. The poached parakeets belong mainly to a quite diverse genetic group of the species and their most likely origin is between northern Michoacan and southern Nayarit, Mexico. We found no evidence that poachers included individuals from Central American international trafficking with individuals from Mexico in the sample.

## Methods

### Genetic diversity

We used the Cytb gene sequences of 80 poached parakeets previously reported by Padilla-Jacobo et al. [21] (GenBank Access: MF441391-MF441470). The samples were obtained from a seizure by Mexican authorities from a vendor in the city of Tlajomulco de Zuñiga, Jalisco, Mexico in August 2014 (Fig. 1). In testimony, the vendor told officers that all individuals came from Sinaloa, Mexico; however, the confession was questioned. Individuals were turned over to the CIVS in Guadalajara, Jalisco, who allowed us to collect biological samples (feather and blood) of 80 *E. canicularis* chicks. In addition, 53 Cytb sequences (GenBank Access: MF441334-MF441390) of parakeets from wild populations distributed on the Pacific slope of Mexico from Sinaloa to Guerrero were used [21] (Fig. 1).

Alignment and construction of data matrices were carried out with PhyDE [47]. Numbers of haplotypes (H) and polymorphic sites (S), nucleotide (Pi) and haplotype (Hd) diversity, and Tajima’s D, and Fu’s F values were determined using ARLEQUIN v3.1 [48].

### Rarefaction and extrapolation curves

We used rarefaction and extrapolation to calculate the richness of the haplotypes in the species using the individual-based method (Coleman method). The application of rarefaction and extrapolation curves is especially useful in the area of ecology and conservation biology to estimate the richness of species in an area [49–53]. Moreover, estimates based on rarefaction and extrapolation curves have been applied to the analysis of DNA sequence analysis, particularly in the estimation of bacterial and sea slug diversity, and to investigate prey preference [54–57]. In this analysis, we assumed that each haplotype represents a “species”, and multiple individual-based input was used to evaluate three groups of the samples (wild, poached, and combined). The curves were estimated based on repeated resampling of 100 replicates without replacement. Extrapolation and rarefaction curves were estimated using EstimateS 9.1.0 software [58].

### Haplotype relationships

To review frequencies of haplotypes and their relationships, a haplotype network was constructed by the median-joining method with NETWORK v5 software [59]. In total, the data matrix included 133 sequences, of which 53 were from individuals in wild populations and 80 were from confiscated individuals.

### Genealogical relationships

The inference of genealogic relationships was accomplished under the criteria of maximum likelihood (ML) and Bayesian inference (BI). The matrix included the 36 haplotypes with 931 characters from this study and a Cytb sequence reported for a specimen identified as *E. canicularis* (EcNCBI hereinafter) (GenBank Access: KJ142251.1). A sequence of *Eupsittula pertinax* was included as an outgroup (GenBank Access: HM640208.1). Molecular evolution models were constructed using jModelTest 2.1.1 [60] and selected using the corrected Akaike Information Criterion (cAIC) values [61]. The best model obtained using this criterion was TrN [62]. The ML and BI reconstructions were performed using RAxML v7.8 [63] and MrBayes v3.2 software [64]. The branches-support values were estimated using a bootstrap analysis (BP) of 500 replicates and posterior probabilities (PP). MrBayes runs were performed using the following parameters: four independent runs of four chains each (one cold chain and three hot chains) for 10 million generations, sampling one tree every 1000 generations. Trees and parameters were summarized after discarding 25% of the data (burn-in). The remaining trees were summarized as a majority consensus tree. Trees were visualized using FigTree v1.4.0 [65].

## Supplementary Information

**Additional file 1 **Haplotypes and variable sites in 931 pb of the Cytb gene from 133 samples of poached and wild *E. canicularis* individuals. Number of haplotypes; alignment site; frequency (n) in wild population, in poached, and total sample; locality of natural distribution; poached.

## Data Availability

The dataset supporting the conclusions in this article is available from the GenBank repository [https://www.ncbi.nlm.nih.gov/] under accession numbers MF441391–MF441470 and MF441334–MF441390.

## Abbreviations

A: Adenine
BI: Bayesian Inference
BP: Bootstrap probability
C: Cytosine
cAIC: Akaike Information Criterion
CI: Confidence interval
Cytb: Cytochrome b
CITES: The Convention on International Trade in Endangered Species of Wild Fauna and Flora
CIVS: Wildlife Research and Conservation Centers
EcNCBI: *E. canicularis* reported in NCBI
G: Guanine
H: Haplotype
Hd: Haplotype diversity
H1 to H36: Haplotypes 1 to 36
HGI: Haplogroup I
HGII: Haplogroup II
HGIII: Haplogroup III
HGIV: Haplogroup IV
H (est): Estimated number of haplotypes
IUCN: International Union for Conservation of Nature Red List of Threatened Species
ML: Maximum Likelihood
n: Number of individuals
Pi: Nucleotide diversity
PP: Posterior probability
S: Polymorphic sites
SD: Standard deviation
T: Thymine
TrN: Tamura and Nei
UNODC: United Nations Office on Drugs and Crime
WWF: World Wildlife Fund

## References

[1] May C (2017). Transnational crime and the developing world.

[2] World Wildlife Fund (WWF)/Dalberg (2012). Fighting illicit wildlife trafficking: A consultation with governments.

[3] United Nations Office on Drugs and Crime (UNODC) (2016). World Wildlife Crime Report: Trafficking in protected species.

[4] Convention on International Trade in Endangered Species of Wild Fauna and Flora (CITES). The CITES Appendices. https://www.cites.org/eng/app/appendices.php. Accessed 03 Feb 2017.

[5] Iñigo-Elias EE, Ramos MA, Robinson JG, Redford KH (1991). The psittacine trade in Mexico. Neotropical wildlife use and conservation.

[6] Collar NJ, Juniper AT, Beissinger SR, Snyder NFR (1992). Dimensions and causes of the parrot conservation crisis. New World parrots in crisis: solutions from conservation biology.

[7] Cantú-Guzmán JC, Sánchez-Saldaña M, Grosselet M, Silva-Gámez J (2007). Tráfico ilegal de pericos en México: una evaluación detallada.

[8] Weston M, Memon M (2009). The illegal parrot trade in Latin America and its consequences to parrot nutrition, health and conservation. Bird Pop.

[9] Forshaw J (1989). Parrots of the world.

[10] Howell SN, Webb S (1995). A guide to the birds of Mexico and northern Central America.

[11] Collar NJ, George LP, Krabbe N, Madroño NA, Naranjo LG, Parker TA, Wege DC (2000). Threatened birds of the Americas.

[12] Ridgely RS, Pasquier RF (1981). The current distribution and status of mainland Neotropical parrots. The conservation of new world parrots.

[13] Stotz D, Fitzpatrick J, Parker T, Moskovits D (1996). Neotropical birds: ecology and conservation.

[14] Monterrubio-Rico TC, Charre-Medellín JF, Pacheco-Figueroa C, Arriaga-Weiss S, de Dios V-LJ, Cancino-Murillo R, Escalona-Segura G, Bonilla-Ruz C, Rubio-Rocha Y (2016). Distribución potencial histórica y contemporánea de la familia Psittacidae en México. Rev Mex Biodivers.

[15] Monterrubio-Rico TC, Villaseñor-Gómez LE, Marín-Togo MC, López-Cordova EA, Fabian-Turja B, Sorani-Dalbon V (2007). Distribución histórica y actual del loro cabeza amarilla (*Amazona oratrix*) en la costa central del Pacífico mexicano: ventajas y limitaciones en el uso de GARP en especies bajo fuerte presión de tráfico. Ornitol Neotrop.

[16] Collar NJ, Boesman P, Kirwan GM, del Hoyo J, Elliott A, Sargatal J, Christie DA, de Juana E (2017). Orange-fronted parakeet (*Eupsittula canicularis*). Handbook of the birds of the world alive.

[17] Diario Oficial de la Federación (DOF) (2010). Norma oficial mexicana NOM-059-SEMARNAT-2010 Protección ambiental - Especies nativas de México de flora y fauna silvestres - Categorías de riesgo y especificaciones para su inclusión, exclusión o cambio - Lista de especies en riesgo.

[18] Procuraduría Federal de Protección al Ambiente (PROFEPA). https://www.gob.mx/profepa/articulos/trafico-ilegal-de-loros-en-mexico. Accessed 20 Jul 2020.

[19] Morales-Pérez L (2005). Evaluación de la abundancia poblacional y recursos alimenticios para tres géneros de psitácidos en hábitats conservados y perturbados de la costa de Jalisco.

[20] Téllez-García L (2008). Abundancia relativa y características del hábitat de anidación del loro cabeza amarilla (*Amazona oratrix*) en diferentes condiciones de conservación de la vegetación.

[21] Padilla-Jacobo G, Monterrubio-Rico T, Cano-Camacho H, Zavala-Páramo MG (2018). Demographic history of the Orange-fronted parakeet (*Eupsittula canicularis*) in Mexico. Ornitol Neotrop..

[22] Frankham R, Ballou JD, Briscoe DA (2004). A primer of conservation genetics.

[23] Ogden R, Dawnay N, McEwing R (2009). Wildlife DNA forensics−bridging the gap between conservation genetics and law enforcement. Endanger Species Res.

[24] Ogden R, Linacre A (2015). Wildlife forensic science: a review of genetic geographic origin assignment. Forensic Sci Int Genet.

[25] Alacs EA, Georges A, FitzSimmons NN, Robertson J (2010). DNA detective: a review of molecular approaches to wildlife forensics. Forensic Sci Med Pathol.

[26] Bandelt HJ, Forster P, Sykes BC, Richards MB (1995). Mitochondrial portraits of human populations using median networks. Genetics..

[27] Avise JC (2009). Phylogeography: retrospect and prospect. J Biogeogr.

[28] Grant W, Bowen B (1998). Shallow population histories in deep evolutionary lineages of marine fishes: insights from sardines and anchovies and lessons for conservation. J Hered..

[29] Adams MS, Villablanca FX, Meeand A, Hall LS (2007). Consequences of a genetic bottleneck in California Condors: A mitochondrial DNA perspective. Series in Ornithology: Condors in the 21st Century.

[30] Baker AJ, Piersma T, Rosenmeier L (1994). Unraveling the intraspecific phylogeography of snots *Calidris canutus*: a progress report on the search for genetic markers. J für Ornithologie.

[31] Mounce HL, Raisin C, Leonard DL, Wickenden H, Swinnerton KJ, Groombridge JJ (2015). Spatial genetic architecture of the critically-endangered Maui parrotbill (*Pseudonestor xanthophrys*): management considerations for reintroduction strategies. Conserv Genet.

[32] Arbeláez-Cortés E, Nyári ÁS, Navarro-Sigüenza AG (2010). The differential effect of lowlands on the phylogeographic pattern of a Mesoamerican montane species (*Lepidocolaptes affinis*, Aves: Furnariidae). Mol Phylogenet Evol.

[33] Barrera-Guzmán AO, Milá B, Sánchez-González LA, Navarro-Sigüenza AG (2012). Speciation in an avian complex endemic to the mountains of middle America (*Ergaticu*s, Aves: Parulidae). Mol Phylogenet Evol.

[34] Cortés-Rodríguez N, Hernández-Banos BE, Navarro-Sigüenza AG, Peterson AT, García-Moreno J (2008). Phylogeography and population genetics of the amethyst-throated hummingbird (*Lampornis amethystinus*). Mol Phylogenet Evol.

[35] Russello M, Stahala C, Lalonde D, Schmidt K, Amato G (2010). Cryptic diversity and conservation units in the Bahama parrot. Conserv Genet.

[36] Caparroz R, Seixas GHF, Berkunsky I, Collevatti RG (2009). The role of demography and climatic events in shaping the phylogeography of *Amazona aestiva* (Psittaciformes, Aves) and definition of management units for conservation. Diversity Distrib.

[37] Presti FT, Guedes NM, Antas PT, Miyaki CY (2015). Population genetic structure in hyacinth macaws (*Anodorhynchus hyacinthinus*) and identification of the probable origin of confiscated individuals. J Hered.

[38] Freeland JR (2005). Molecular Ecology.

[39] Chassaing O, Desse-Berset N, Duffraisse M, Piquès G, Hänni C, Berrebi P, Williot P, Rochard E, Desse-Berset N, Kirschbaum F, Gessner J (2011). Palaeogeographic patterns of *A. sturio*. Biology and Conservation of the European Sturgeon *Acipenser sturio* L 1758.

[40] Padilla-Jacobo G, Cano-Camacho H, López-Zavala R, Cornejo-Pérez ME, Zavala-Páramo MG (2018). Evolutionary history of Mexican domesticated and wild *Meleagris gallopavo*. Genet Sel Evol.

[41] Padilla-Jacobo G, Monterrubio-Rico TC, Cano-Camacho H, Zavala-Páramo MG (2016). Use of phylogenetic analysis to identify evolutionarily significant units for the Orange-fronted parakeet (*Eupsittula canicularis*) in Mexico. Ornitol Neotrop..

[42] Araki H, Cooper B, Blouin MS (2007). Genetic effects of captive breeding cause a rapid, cumulative fitness decline in the wild. Science..

[43] Zeng Y, Jiang Z, Li C (2007). Genetic variability in relocated Père David’s deer (*Elaphurus davidianus*) populations-implications to reintroduction program. Conserv Genet.

[44] Frankham R (2008). Genetic adaptation to captivity in species conservation programs. Mol Ecol.

[45] Smith S, Hughes J (2008). Microsatellite and mitochondrial DNA variation defines island genetic reservoirs for reintroductions of an endangered Australian marsupial, Perameles bougainville. Conserv Genet.

[46] Hedrick PW, Fredrickson R (2008). Captive breeding and the reintroduction of Mexican and red wolves. Mol Ecol.

[47] Müller J, Müller K, Neinhuis C, Quandt D. PhyDE-Phylogenetic data editor. http://www.phyde.de/index.html. Accessed 13 Mar 2015.

[48] Excoffier L, Laval G, Schneider S (2005). Arlequin (version 3.0): an integrated software package for population genetics data analysis. Evol Bioinform Online.

[49] Colwell RK, Coddington JA (1994). Estimating terrestrial biodiversity through extrapolation. Philos Trans R Soc Lond Ser B Biol Sci.

[50] Colwell RK, Mao CX, Chang J (2004). Interpolating, extrapolating, and comparing incidence-based species accumulation curves. Ecology..

[51] Chao A, Kotz S, Balakrishnan N, Read CB (2005). Species estimation and applications. Encyclopedia of statistical sciences.

[52] Chao A, Colwell RK, Lin C-W, Gotelli NJ (2009). Sufficient sampling for asymptotic minimum species richness estimators. Ecology..

[53] Colwell RK, Chao A, Gotelli NJ, Lin S-Y, Mao CX, Chazdon RL, Longino JT (2012). Models and estimators linking individual-based and sample-based rarefaction, extrapolation and comparison of assemblages. J Plant Ecol.

[54] Feng B-W, Li X-R, Wang J-H, Hu Z-Y, Meng H, Xiang L-Y, Quan Z-X (2009). Bacterial diversity of water and sediment in the Changjiang estuary and coastal area of the East China Sea. FEMS Microbiol Ecol.

[55] Wilson NG, Schrödl M, Halanych KM (2009). Ocean barriers and glaciation: evidence for explosive radiation of mitochondrial lineages in the Antarctic Sea slug *Doris kerguelenensis* (Mollusca, Nudibranchia). Mol Ecol.

[56] Salinas-Ramos VB, Herrera Montalvo LG, León-Regagnon V, Arrizabalaga-Escudero A, Clare EL (2015). Dietary overlap and seasonality in three species of mormoopid bats from a tropical dry forest. Mol Ecol.

[57] Ibáñez C, Popa-Lisseanu AG, Pastor-Beviá D, García-Mudarra JL, Juste J (2016). Concealed by darkness: interactions between predatory bats and nocturnally migrating songbirds illuminated by DNA sequencing. Mol Ecol.

[58] Colwell RK. EstimateS: statistical estimation of species richness and shared species from samples. Version 9, Software. http://purl.oclc.org/estimates. Accessed 15 Jan 2017.

[59] Fluxus Technology Ltd. Network 5. http://www.fluxus-engineering.com/sharenet.htm. Accessed 25 Jan 2017.

[60] Posada D (2008). jModelTest: phylogenetic model averaging. Mol Biol Evol.

[61] Alfaro ME, Huelsenbeck JP (2006). Comparative performance of Bayesian and AIC-based measures of phylogenetic model uncertainty. Syst Biol.

[62] Tamura K, Nei M (1993). Estimation of the number of nucleotide substitutions in the control region of mitochondrial DNA in humans and chimpanzees. Mol Biol Evol.

[63] Stamatakis A (2014). RAxML version 8: a tool for phylogenetic analysis and post-analysis of large phylogenies. Bioinformatics..

[64] Ronquist F, Huelsenbeck JP (2003). MrBayes 3: Bayesian phylogenetic inference under mixed models. Bioinformatics..

[65] Rambaut A. FigTree v1.4.0. http://tree.bio.ed.ac.uk/software/figtree/. Accessed 16 Mar 2015.

